# Aetiology of maternal mortality using verbal autopsy at Sokoto, North-Western Nigeria

**DOI:** 10.4102/phcfm.v5i1.442

**Published:** 2013-06-20

**Authors:** Jamila A. Garba, Sadiq Umar

**Affiliations:** 1 Department of Community Health, Universiti Putra Malaysia, Malaysia; 2 Department of Community Health, Usmanu Danfodiyo University, Nigeria

## Abstract

**Background:**

Maternal mortality in developing countries is higher than that in developed countries. There are few published articles on the factors associated with maternal deaths in northern Nigeria.

**Objectives:**

The objective of this study was to identify the medical causes and factors associated with maternal mortality in Sokoto, northern Nigeria.

**Method:**

A verbal autopsy questionnaire was used to interview close relatives of women within the reproductive age group who had died of pregnancy-related complications in the Sokoto metropolis during the preceding two years. A multistage sampling method using simple random sampling at each step was used to select areas of study within the Sokoto metropolis. Data analysis was carried out using a statistical package for social sciences (SPSS), version 19, and the Spearman correlation was used to test association. Significance level was set at 0.05.

**Results:**

The major causes of death were haemorrhage (48.3%), eclampsia (19%) and prolonged labour (13.8%). The association between maternal mortality and the absence of antenatal booking was significant (*p* < 0.001); the association between maternal mortality and the ‘three delays’ was also significant (*p* = 0.013). The association between maternal mortality and educational status and occupation was, however, not significant (*p* = 0.687 and *p* = 0.427 respectively).

**Conclusion:**

The medical causes of maternal mortality identified in this study were similar to those of the hospital-based studies in the area. In addition, an association between maternal deaths and the ‘three delays’ and the absence of antenatal booking was found. There is a need for public education efforts to address these factors in order to reduce maternal mortality in the study area.

## Abstrait

**Étiologie de la mortalité maternelle en utilisant l'autopsie verbale à Sokoto, Nigeria du Nord-Ouest**

**Présentation:**

Dans les pays en voie de développement, la mortalité maternelle est supérieure aux taux observés dans les pays développés. Peu d'articles consacrés aux facteurs associés aux décès maternels au Nord du Nigeria sont publiés.

**Objectifs:**

L'objectif de cette étude était d'identifier les causes et facteurs médicaux associés à la mortalité maternelle à Sokoto, au Nord du Nigeria.

**Méthode:**

Un questionnaire d'autopsie verbale a été utilisé afin d'interroger les parents proches des femmes en âge de procréer et décédées suite à des complications associées à la grossesse dans la ville de Sokoto au cours des deux années précédentes. Une méthode d’échantillonnage à plusieurs degrés utilisant un échantillonnage aléatoire simple à chaque étape a été utilisée afin de sélectionner des domaines d’étude dans la ville de Sokoto. L'analyse des données a été réalisée en utilisant le logiciel Statistical Package for Social Science (SPSS), version 19, et la corrélation de Spearman a été utilisée afin de tester l'association. Le seuil de signification a été fixé à 0,05.

**Résultat:**

Les principales causes de décès étaient: hémorragie (48.3%), éclampsie (19%) et travail prolongé (13.8%). L'association entre mortalité maternelle et absence de suivi anténatal était importants (*p* < 0.001); l'association entre mortalité maternelle et les « trois délais » était également importante (*p* = 0.013). L'association entre mortalité maternelle et niveau d'instruction et professionnel n’était cependant pas importante (respectivement *p* = 0.687 et *p* = 0.427).

**Conclusion:**

Les causes médicales de la mortalité maternelle identifiées dans cette étude étaient similaires à celles des études basées en hôpital dans la région. De plus, une association entre les décès maternels et les « trois délais » et l'absence de suivi anténatal a été notée. Il est nécessaire que des efforts d’éducation du public soient réalisés afin de traiter ces facteurs et réduire la mortalité maternelle dans la zone de l’étude.

## Introduction

### Problem statement

One of the key indicators of the health status of a nation is maternal mortality.^1^ Knowing the underlying causes of these deaths is the cornerstone in planning preventive measures to reduce maternal mortality.

#### Key focus

In countries where vital registration data are scarce and most deaths occur at home, verbal autopsy is used to assess the circumstances surrounding the deaths.^1, 2^ Verbal autopsies will also identify multiple contributing factors to maternal deaths so that appropriate intervention can be instituted.

#### Trends

Maternal mortality is defined as the death of a woman whilst she is pregnant or within 42 days of the termination of her pregnancy, irrespective of the duration and site of the pregnancy, from any cause related to or aggravated by the pregnancy or its management but not related to accidental or incidental causes.^3^ The risk of a woman in a developing country dying of complications resulting from pregnancy is about 36 times higher than that of a woman in a developed country.^4^

The estimate for maternal mortality in 2008 was 358 000 maternal deaths globally, 99% of these deaths occurring in developing countries.^3^ Sub-Saharan Africa accounted for 204 000 of these deaths, whilst South Asia had 109 000 maternal deaths.^3^

In 2008 Nigeria ranked second amongst the countries with high maternal mortality, with an estimate of 50 000 maternal deaths; in developed countries this number was as low as five maternal deaths or less.^3^ In Sokoto a hospital-based study has shown a high maternal mortality ratio of 2151 per 100 000 live births.^5^

The causes of maternal mortality can be direct or indirect obstetric causes, but maternal mortality can also be associated with non-medical factors.^1, 6^ These non-medical factors can be social, cultural or economic.1 Social factors refer to ethnicity, religion, educational status, economic status, occupation, family and political system, whilst relevant cultural factors are the attitudes, values and belief systems that are common in a community. Identifying the causes and associated factors of maternal mortality is important when planning intervention measures for reducing the mortality rate. The associated factors may not be revealed by a hospital-based study as a number of deaths in developing countries occur outside the hospitals and are not registered.^6^ Verbal autopsy is used to obtain information regarding maternal mortality, and a close relative who is knowledgeable about the circumstances surrounding the death is interviewed.^1, 6^ The interview gives detailed information about the events, apart from medical causes, that led to the death. Verbal autopsy has been used in various countries for the assessment of maternal mortality.^7–14^ Most studies in Nigeria, however, are hospital-based studies.^5, 15–17^

After identifying the medical causes, the ‘three delay model’ was used in this study to identify some of the factors associated with maternal mortality. Delay 1 was a delay in making the decision to seek hospital care, delay 2 was a delay in reaching the hospital and delay 3 was a delay in receiving adequate care at the hospital.^18^

#### Objectives

The objective of this study was to determine the medical causes and factors associated with maternal mortality.

#### Contribution to the field

Identifying the associated factors in addition to the medical causes of maternal mortality will guide decision makers when they plan preventive measures to decrease maternal mortality in a region.

## Research method and designs

### Materials

The study population consisted of relatives of women in the reproductive age group (15–49 years) who had died of a pregnancy-related condition during the previous two years.

### Setting

Sokoto is the state capital of Sokoto state which lies in the extreme north-western part of Nigeria. The city is situated on longitudes 11°30′–13°50′E and latitudes 4°–6°40′N. It covers a land area of 26 648.48 km^2^. The Sokoto metropolis comprises Sokoto South and Sokoto North local governments and some parts of Dange-shuni and Wamakko local governments. Sokoto state is estimated to have a total population of 3 702 676 (1 863 713 males and 1 838 963 females) and 430 698 of these live in the Sokoto metropolis (276 767 of whom are females).^19^ The percentage of women within the reproductive age group is 27.5%, the female literacy level in any language is 42%, the percentage of unemployed and underemployed females is 41% and the percentage of pregnant women who had registered for antenatal care in 2007 was 42%.^20^

Sokoto North local government has 15 wards and 38 areas in all the wards, Sokoto South has 12 wards and 32 areas in all the wards, Dange-shuni has 10 wards with a total of 22 areas whilst Wamakko has 10 wards with a total of 20 areas.^21^ The major tribe there is the Hausa-Fulani but other minor tribes such as Gobirawa, Zabarmawa, Kabawa and Adarawa are also found there. The major religion is Islam and a few inhabitants are Christians.^22^

### Design

The study was a descriptive cross-sectional study using an interviewer administered verbal autopsy questionnaire containing closed and open-ended questions. The interviews were conducted at the homes of the respondents, who were all close relatives of the deceased. The sampling method used in selecting the areas of study was a multistage sampling method using simple random sampling at each stage. Four wards were randomly selected from Sokoto North, and twelve areas were then selected from these wards. Three wards and nine areas were selected in a similar way from Sokoto South, two wards and six areas from Dange-shuni, and two wards and six areas from Wamakko. All the households in the selected areas were visited and enquiries made regarding maternal deaths in the preceding two years.

### Procedure

Ten field assistants were trained to conduct the interview in the selected areas. The ethics approval was forwarded to the District Heads and religious leaders in the selected areas and their informed consent was obtained. They also supplied the names and locations of the maternal deaths in the preceding two years and a house-to-house visit was performed in all the selected areas. Close relatives and neighbours who were knowledgeable about the events surrounding the deaths of the mothers were interviewed. The respondents were asked about the reproductive history of the deceased, complications during the pregnancy that had been observed prior to her death and the socio-cultural and economic factors that might have contributed to her death. The verbal autopsy questionnaire also included questions about the symptoms she had had before her death, the time when the problem had first been recognised, when the decision to seek care had been reached, how she had arrived at the right health centre, how long it took her to reach the health centre, the time she arrived there, the time she received care and whether she received appropriate care or not. An open-ended question was also asked to obtain any other information about her death.

### Analyses

Data analysis was performed using a statistical package for social sciences (SPSS), version 19. The alpha level of significance was set at 0.05. The dependent variable was maternal death whilst the independent variables were socio-demographic characteristics, antenatal booking, parity and the ‘three delays’. The probable medical causes of death and the age of the deceased were obtained in percentages and are presented in a pie chart.

The educational status, occupation, booking status, parity and delays were presented in percentages. A test of association between maternal mortality and educational status, occupation, booking status, parity and the ‘three delays’ was carried out using the non-parametric Spearman correlation.

## Results

**FIGURE 1:**
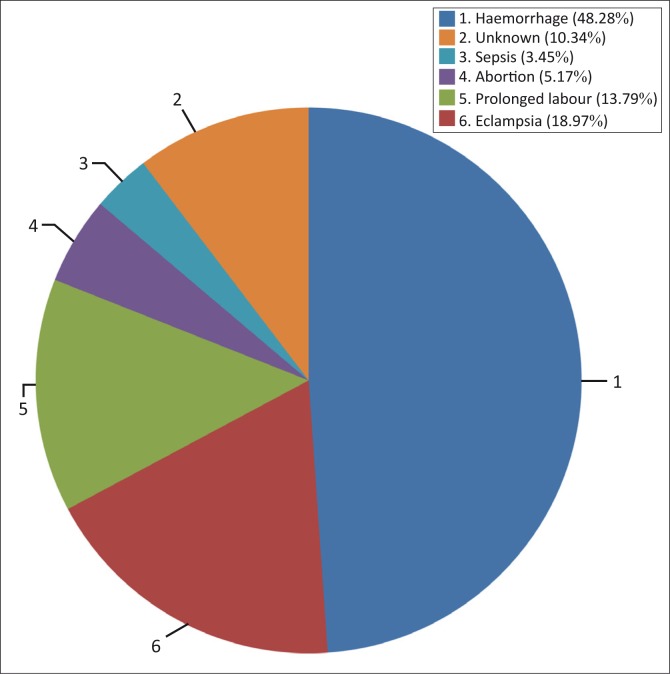
The medical causes of maternal mortality.

Sixty-two maternal deaths were identified in the selected areas and verbal autopsy questionnaires were filled in for 58 of those. Detailed information could not be obtained in four of the cases. The number of deaths caused by haemorrhage were 28 (48.3%) and those caused by eclampsia were 11 (19%). There were 8 (13.8%) deaths after prolonged labour, 3 (5.2%), following abortion and 2 (3.4%) caused by sepsis. In 6 cases (10.3%) the cause of death was unknown (Figure 1). The specific types of haemorrhage and abortion could also not be identified.

**FIGURE 2:**
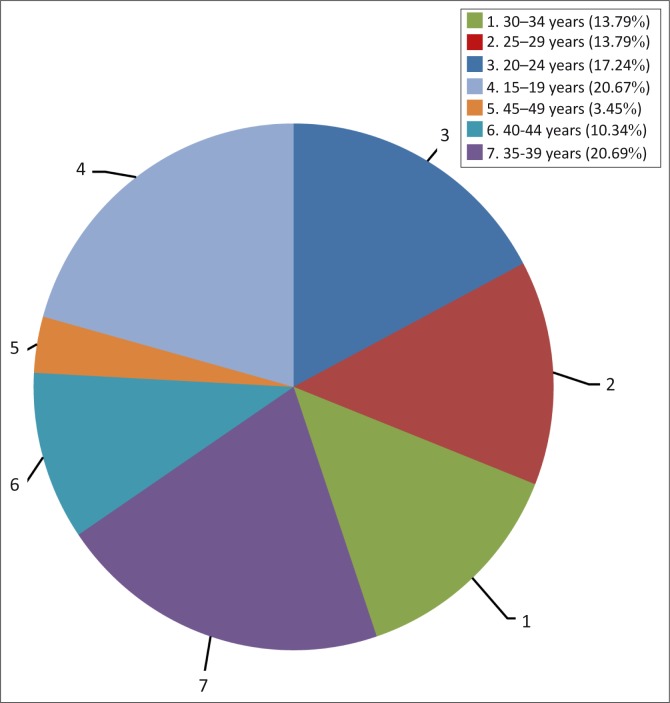
Age group distribution of the deceased.

The age groups with the highest number of maternal deaths were 15–19 years and 35–39 years and there were 12 deaths in each group (20.7%). This was followed by the age group 20–24 years, in which there were 10 (17.2) deaths (Figure 2). Thirty-one (53.4%) women had received informal education whilst 27 (46.6%) had had formal education (Table 1). The association between maternal mortality and educational status was not significant, however, with *p* = 0.687 (Table 2). Forty (69%) women had been housewives and 8 (13.8%) had been civil servants, as shown in Table 1. There was no significant association between maternal death and occupation, with *p* = 0.427 (Table 2). Thirty-three (56.9%) of the women had not been booked for antenatal care, whilst 25 (43.1%) had been booked (Table 1); the association between maternal mortality and lack of antenatal booking was significant, with *p* < 0.001 (Table 2). The highest number of maternal deaths occurred in primigravida (24 or 41.4%). This was followed by grand multipara (19 or 32.8%) and multipara (15 or 25.9%) (Table 1). However, no significant association between maternal death and parity was found, with *p* = 0.061 (Table 2). The main medical causes of death in primipara were eclampsia (*n* = 7, 29.2%) and prolonged labour (*n* = 7, 29.2%) whilst the main cause of maternal death in grand multipara was haemorrhage (*n* = 11, 57.9%) (Table 3).

**TABLE 1:** Percentage distribution of the independent variables.

Variable	*n*	%
**Parity**		
Primipara	24	41.4
Multipara	15	25.9
Grand multipara	19	32.8
**Booking status**		
Unbooked	33	56.9
Booked	25	43.1
**Educational status**		
Primary	6	10.3
Secondary	13	22.4
Tertiary	8	13.8
Informal	31	53.4
**Occupation**		
House wife	40	69
Civil servant	8	13.8
Others	10	17.2
**Place of delivery**		
Home	46	79.3
Hospital	7	12.1
Undelivered	5	8.6
**Place of death**		
Home	37	63.8
Hospital	21	36.2
**Delays**		
Delay 1	42	72.4
Delay 2	6	10.3
Delay 3	6	10.3
Unknown	4	6.9

**TABLE 2:** Association of independent variables with maternal mortality.

Variable	*r*	*p*-value
Educational status	0.054	0.687
Occupation	0.106	0.427
Antenatal booking	0.469	< 0.001
Parity	0.284	0.061
The ‘three delays’	0.323	0.013

*r*, Spearman rho; *p*-value, 5% level of significance.

**TABLE 3:** Medical causes of death by parity.

Parity	Medical cause of death	*n*	%
Primipara	Eclampsia	7	29.2
	Prolonged labour	7	29.2
	Haemorrhage	5	20.8
	Abortion	3	12.5
	Unknown	2	8.3
Multipara	Haemorrhage	8	53.3
	Prolonged labour	3	20
	Eclampsia	2	13.3
	Sepsis	1	6
	Unknown	1	6
Grand multipara	Haemorrhage	11	57.9
	Prolonged labour	2	10.5
	Eclampsia	2	10.5
	Sepsis	1	5.3
	Abortion	1	5.3
	Unknown	2	10.5

The other associated factors were analysed in terms of the ‘three delays’. Delay 1 is a delay in making the decision to seek hospital care and there were 42 cases (72.4%) with that form of delay (Table 1). Delay 2 is a delay in reaching the hospital; there were six cases (10.3%) with that form of delay (Table 1). Delay 3 is a delay in receiving adequate care at the hospital and there were six cases (10.3%) with that form of delay (Table 1). In four cases (6.9%) no form of delay was identified, however. There was a significant association between maternal mortality and the ‘three delays’ and there were significantly more cases of delay 1, with *p* = 0.013 (Table 2).

## Ethical Considerations

Approval for doing the study was obtained from the Ethics Committee of Usmanu Danfodiyo University Teaching Hospital.

### Potential benefits and hazards

The study was not hazardous to the deceased or their relatives. A potential benefit of the study is that the information obtained can be used to plan preventive measures for the relatives and the nation at large regarding maternal mortality.

### Recruitment procedures

Informed consent was obtained from the district head and religious leaders in each selected area. A respondent's information sheet, written in the local language, was given to the relatives and informed consent was obtained. Confidentiality of the information obtained was also assured.

## Discussion

This study showed that the major medical causes of maternal mortality were haemorrhage, eclampsia and prolonged labour. This corresponds to the previous hospital-based study in Sokoto which had identified the major causes of death to be ruptured uterus, haemorrhage, eclampsia and infection.^5^ Ruptured uterus was not identified in this study because the respondents lacked the medical knowledge to give that diagnosis, and cases of ruptured uterus would probably have been classified under haemorrhage. The causes of maternal mortality revealed by this study are similar to those identified in northern central Nigeria,^23^ north-eastern Nigeria,^15^ and some southern states in Nigeria.^16, 17^ There are variations in the order of frequency in which they occurred, however.

The age groups with the highest number of maternal deaths were 15–19 years and 35–39 years. This is consistent with the findings regarding cause of death: the age group of 15–19 years were primigravida who had mostly died of eclampsia and prolonged labour, whilst the 35–39 year olds were grand multipara who had mostly died of haemorrhage. Similar findings were also noted in the northern central part of Nigeria in which the majority of the deaths occurred in the group ≤ 15 and ≥ 40 years.^18^ However, it is not consistent with the finding in the north-eastern part of Nigeria where there was no difference between the age groups.^15^ The variation may be due to a difference in the form of data collection and the number of maternal deaths obtained.

This study also found that maternal deaths in those that had not been booked for antenatal care were more than in those who had been booked and that there was a statistical significance in the difference between the booked and unbooked (*p* < 0.001). This indicates that those that are booked are less likely to die of pregnancy-related complications. Similar findings were also found in north-eastern Nigeria^15^ and the south-eastern^16^ and northern central parts of Nigeria.^18^

The statistical non-significant association that was obtained between the educational status and occupation of the maternal deaths may be because of the small number of maternal deaths obtained from this study. However, it may also be because of the prevalent cultural practice that stipulates that a woman cannot make any decision on her own and without interference from the elders, regardless of her educational status or occupation.

The statistical significant association observed between maternal mortality and the ‘three delays’ has shown that these delays may contribute to the high maternal mortality in this region. Some experienced more than one form of delay. There was a similar finding in Argentina where delay 1 was identified as the most frequent,^8^ whilst in Gambia^10, 19^ and India^12^ delay 3 was the most common. The variation in results may be caused by the relatives not being aware of some of the delays occurring at the hospital.

In this study, the reasons given for delay 1 were that the people involved were unable to identify the early signs and symptoms of complications and did not realise that there was a need for hospital care. Most deliveries are conducted at home by experienced women or traditional birth attendants and they lack the medical knowledge to recognise symptoms of potentially life-threatening conditions. In some cases the decision maker in the family had not been present and there was some delay before he could be reached and asked for permission to go to the hospital. This decision maker may not necessarily be the husband. After the decision to go to the hospital had been made, there could have been further delays because of long distances and the absence of an efficient transport system to the appropriate hospital. In some cases there was a delay because of financial constraints, because health care is not free. Even in the hospital, there were further delays because of the lack of money to buy the necessary materials to initiate care. Because blood donation is not free, relatives have to donate blood before the patient can receive blood. Some of the reasons for the delays at the hospital may not have been known to the relatives.

## Limitations of the study

The reason why only a small number of cases were used in this study was because only one quarter of the areas in the Sokoto metropolis were randomly selected; hence, a wider study covering all maternal deaths in the metropolis within the study period would be better for generalisation. There were also memory recall problems and so it was not always easy to obtain adequate histories; the problem was partly solved by using more than one person with information regarding the deceased to answer the questionnaire. There was also a problem when the exact medical cause of death had to be ascertained as the respondents lacked medical knowledge. The study could not identify some of the reasons for delays occurring at the hospital.

### Recommendations

It is recommended that a wider study be performed in order to cover all the maternal deaths in the area within the specified period of the study. It is also recommended that there be verbal autopsies for all hospital deaths so that other associated factors relating to maternal mortality can be identified in addition to hospital factors and medical causes.

There should be a regular health education programme to educate the community about antenatal booking and hospital delivery so that the delay in decision making can be avoided. Another option is to educate the traditional birth attendants so that they can recognise early signs of life-threatening conditions. Improvement in the transportation services for emergencies is important. The service for handling emergency obstetrics should be made free or subsidised and blood transfusion services should be readily available at all times.

## Conclusion

This study has identified the probable medical causes of maternal mortality in Sokoto metropolis to be haemorrhage, eclampsia and prolonged labour. There was significant association between maternal mortality and the absence of antenatal booking and the ‘Three delays’. The main delay that contributed to maternal death was a delay in making the decision to seek hospital care. Hence, there is a need to educate important role players in communities so that these kinds of delays can be minimised.
